# RNA Profiles of the Korat Chicken Breast Muscle with Increased Carnosine Content Produced through Dietary Supplementation with β-Alanine or L-Histidine

**DOI:** 10.3390/ani11092596

**Published:** 2021-09-03

**Authors:** Satoshi Kubota, Kasarat Promkhun, Panpradub Sinpru, Chanadda Suwanvichanee, Wittawat Molee, Amonrat Molee

**Affiliations:** School of Animal Technology and Innovation, Institute of Agricultural Technology, Suranaree University of Technology, Nakhon Ratchasima 30000, Thailand; kasarat2862@gmail.com (K.P.); panpradub.s@g.sut.ac.th (P.S.); suwanvit.chanadda@gmail.com (C.S.); wittawat@sut.ac.th (W.M.); amonrat@sut.ac.th (A.M.)

**Keywords:** RNA-Seq, slow-growing chicken, breast meat, carnosine content, meat toughness

## Abstract

**Simple Summary:**

Carnosine is a bioactive food component with several potential health benefits for humans due to its physiological functions. Dietary supplementation with β-alanine or L-histidine can increase the carnosine content of skeletal muscles in chickens. Dietary supplementation with β-alanine or L-histidine has produced a slow-growing chicken variety with high carnosine content in the breast meat; however, the supplementation with L-histidine alone softens the meat toughness, which may affect consumers’ willingness to buy the meat. Gene expression is a key factor that influences meat quality. Understanding the molecular mechanisms that affect carnosine content and meat toughness would allow the production of more value-added slow-growing chickens. We compared global gene expression in chicken breast muscles with differing carnosine contents and meat toughness produced through dietary supplementation with β-alanine or L-histidine. We identified differentially expressed genes involved in regulating myosin, collagen, intramuscular fat, and calpain—factors that may affect meat tenderness. Pathway enrichment analysis indicated that the insulin-related and adipocytokine signaling pathways were altered by dietary supplementation with β-alanine or L-histidine. These data will be useful for future studies on carnosine content and meat toughness in slow-growing chickens.

**Abstract:**

Korat chicken (KRC) is a slow-growing chicken bred in Thailand, whose meat exhibits a unique toughness. A previous study produced KRC breast meat containing high carnosine content through dietary supplementation with β-alanine or L-histidine; however, the KRC that were fed an L-histidine-supplemented diet produced meat that was significantly more tender. Herein, we performed RNA-Seq to identify candidate genes involved in the regulation of carnosine content and meat toughness. Total RNA was isolated from five female KRC breast muscles in each treatment group that KRC fed diets without supplementation, supplemented with β-alanine or L-histidine. Compared to the non-supplemented group, we identified 118 and 198 differentially expressed genes (DEGs) in the β-alanine or L-histidine supplementation groups, respectively. Genes potentially related to meat tenderness—i.e., those regulating myosin, collagen, intramuscular fat, and calpain—were upregulated (*LOC107051274*, *ACSBG1*, and *CAPNS2*) and downregulated (*MYO7B*, *MYBPH*, *SERPINH1*, and *PGAM1*). However, carnosine synthase gene was not identified. Functional enrichment analysis identified pathways affected by dietary supplementation, including the insulin signaling pathway (β-alanine supplementation) and the insulin resistance and adipocytokine signaling pathways (L-histidine supplementation). The FoxO signaling pathway was identified as a regulatory network for both supplementation groups. The identified genes can be used as molecular markers of meat tenderness in slow-growing chickens.

## 1. Introduction

Korat chicken (KRC) is produced by crossing the males of Thai indigenous chickens with the female Suranaree University of Technology (SUT) breeder line, and has a slow growth rate. KRC meat contains lower levels of fat and higher quantities of collagen compared to the meat from a commercial broiler. It also has a firm and chewy texture, which attracts domestic consumers to a greater extent than broiler meat in Thailand [1]. However, female KRCs are not as preferred by farmers because they grow at a slower rate than males, but have the same selling price. Thus, adding more value to female KRCs is desirable. Intarapichet and Maikhunthod [2] reported that meat from Thai indigenous female chickens contains higher levels of carnosine than meat from males. Carnosine is well-known for its physiological functions, and intake of carnosine has been shown to contribute to human health in many age-associated diseases and conditions [3,4]. Carnosine concentration in the skeletal muscles of broiler chickens can be enhanced by dietary supplementation with the constituent amino acids of carnosine (i.e., β-alanine and L-histidine) [5,6]. Suwanvichanee et al. [7] reported that compared to KRCs fed a diet without supplementation, female KRCs fed a diet supplemented with β-alanine or L-histidine have significantly higher carnosine content in breast muscles (by 26.42 and 32.76%, respectively). Growth and slaughtering performances were not significantly different between dietary treatments; however, meat toughness (as measured by shear force value) was significantly reduced only by supplementation with L-histidine [7]. These findings suggest that female KRCs with increased carnosine content produced through supplemented diets can increase the consumers’ demand as a functional food, which motivates farmers’ willingness to raise them. However, dietary supplementation with L-histidine also reduces the toughness of the meat, which is a unique property of KRC meat.

Among the traits that strongly affect the quality of meat and consumers’ willingness to purchase poultry products, tenderness is the most important attribute [8]. Many factors contribute to the wide variation in tenderness of poultry meat, including bird strain, age, sex, environment, antemortem and/or postmortem handling conditions, and cooking method [9]. Several studies have identified the genetic basis of tenderness—including genes encoding the proteins of the calpain system (*CAPN1*)—in broiler chickens [10]. Using a whole transcriptomic analysis, Piórkowska et al. [11] identified biological processes and genes that are potentially associated with factors affecting chicken breast meat tenderness, such as degradation of filamins (*ASB2*), lipogenesis (*PLIN1* and *THRSP*), and collagen synthesis (*COL16A1*, *COL20A1*, *LEPREL4*, *PCOLCE2*, *P4HA3*, and *VWA1*). These findings suggest that such candidate genes can be used as markers to determine meat tenderness; however, it is unclear how the actions of these genes are affected in chickens fed a supplemented diet to increase carnosine content.

Transcriptome sequencing technology (RNA-Seq) can help elucidate the genetic and physiological processes that regulate the phenotype of particular quantitative traits [12]. Dietary supplementation has been shown to alter the transcriptomic profile of breast muscles in chickens, and RNA-Seq can be used to identify key genes and molecular regulatory mechanisms related to factors affecting meat quality, such as color and taste [13], and melanin deposition [14]. In this study, we performed RNA-Seq in female KRC breast muscle to identify differentially expressed genes and underlying molecular mechanisms affected by the diets supplemented with β-alanine or L-histidine. To the best of our knowledge, this is the first study on global gene expression in chicken breast muscle with differing carnosine contents and meat toughness produced through dietary supplementation. The identified genes can be used as a molecular marker associated with carnosine content and meat tenderness in slow-growing chickens.

## 2. Materials and Methods

### 2.1. Animals and Sample Collection

All animal procedures applied in this research were approved by the Ethics Committee on Animal Use of the Suranaree University of Technology (user application ID: U1-08780-2563).

In this study, we used the 15 breast muscles of female KRCs collected in a previous study by Suwanvichanee et al. [7]. Briefly, a total of 300 one-day-old female KRCs were generated by crossbreeding male Thai indigenous chickens and the female SUT breeder line. All chicks were reared in open-sided housing from 0 to 3 weeks of age. KRC feeding management is designed as three different phases based on the age of the chicken: 0 to 3, 4 to 6, and 7 to 10 weeks. The basal diet at the initial, second, and final phases contains 21, 19, and 17% of protein, respectively. Birds were raised on a basal diet at the initial phase before starting the experiments. Chickens were fed an experimental diet containing β-alanine (Sigma-Aldrich, St. Louis, MO, USA, 146064) or L-histidine (AppliChem GmbH, Darmstadt, Germany, A3738) from 4 to 10 weeks of age to increase carnosine content. Group A was the control without supplements. The other groups were fed by diets supplemented with 1% (1 g/100 g diet) β-alanine (B) or 0.5% (0.5 g/100 g diet) L-histidine (C). Each treatment group contained five replicates (cages) with 20 chickens per replicate. All chickens were given feeds and water ad libitum during the experiment. The composition of the experimental diet is presented in Table S1. At the end of the feeding trial, seven chickens were removed from each cage. Of these seven chickens, five were fasted and euthanized for carcass and meat quality measurements and the other two chickens were sacrificed for carnosine content measurements. For transcriptome analysis, a small amount of the left breast muscle tissue was extracted from similar parts of the two birds used for the carnosine content measurement. The muscle tissue was collected in tubes, frozen in liquid nitrogen immediately, and kept at −80 °C until the usage for transcriptome analysis. 

### 2.2. RNA Extraction

The breast muscle from one of the two KRCs was subjected to RNA extraction. Total RNA was extracted from the 15 chickens—i.e., one chicken from each replicate of each treatment group—using TRIzol Reagent (Thermo Fisher Scientific, Waltham, MA, USA) following the manufacturer’s protocol. The purity of the extracted RNA was analyzed using 1% agarose gel electrophoresis and measuring the absorbance using a NanoDrop 2000 spectrophotometer (Thermo Fisher Scientific). The RNA integrity number (RIN) was verified using an Agilent 2100 Bioanalyzer (Agilent Technologies, Santa Clara, CA, USA) and 1 µg of total RNA with RIN ≥ 6.7 was used for subsequent transcriptome analysis. 

### 2.3. Library Preparation and Sequencing

Library preparation, sequencing, and analysis were performed by Vishuo Biomedical (Thailand) Ltd. according to the manufacturer’s instructions. Poly (A) mRNA was isolated using the Poly (A) mRNA Magnetic Isolation Module (NEB, County Road Ipswich, MA, USA) or the VAHTS mRNA-seq V3 Library Prep Kit for Illumina (Vazyme, Nanjing, China). A first-strand synthesis reaction buffer and random primers were applied for mRNA fragmentation and priming. First-strand and second-strand cDNAs were synthesized respectively using ProtoScript II Reverse Transcriptase (NEB) or Second Strand Synthesis Enzyme Mix (NEB). The double-stranded cDNA was then purified using beads. Following DNA repair and dA-tailing using the End Prep Enzyme Mix (NEB), an adaptor was added to both ends by T-A ligation. Adaptor-ligated DNA was then selected using beads, and fragments of ~400 bp with an insert size of approximately 300 bp were recovered. The size-selected DNA was amplified by bridge PCR using the P5 and P7 primers that contain an index for multiplexing. The PCR products were purified using beads, validated, and quantified using a Qsep100 system (BiOptic, New Taipei City, Taiwan) or Qubit 3.0 fluorometer (Invitrogen, Carlsbad, CA, USA), respectively. Libraries were analyzed using an Illumina Novaseq 6000 instrument (Illumina, San Diego, CA, USA) according to the manufacturer’s instructions. Sequencing was performed using a 2 × 150 bp paired-end configuration. Base calling was performed using the NovaSeq software.

### 2.4. Sequencing Quality Assessment and Differential Expression Analysis 

RNA-Seq reads were obtained from the breast muscles of KRC fed the experimental diets. Low-quality and technical sequences such as adapters and primers were removed from the raw sequencing reads using Cutadapt [15] version 1.9.1, and high-quality data (clean reads) were generated for further analysis. Hisat2 [16] version 2.0.1 was used to align the clean reads against the chicken reference genome, GRCg6a (GenBank Assembly ID: GCA_000002315.5). Gene expression levels were estimated based upon fragments per kilobase of transcript per million fragments mapped (FPKM) using HTSeq [17] version 0.6.1. The DESeq2 Bioconductor package [18] was used for differential expression analysis between the control group and each group fed with dietary supplements. The Benjamini–Hochberg method was applied to control the false discovery rate. Transcripts with adjusted values of *p* < 0.05 and an absolute fold change of ≥1.5 were considered to be differentially expressed. The raw data conferred in this publication have been deposited in NCBI’s Gene Expression Omnibus which can be access through GEO Series accession number GSE179643 (https://www.ncbi.nlm.nih.gov/geo/query/acc.cgi?acc=GSE179643, accessed on 7 July 2021).

### 2.5. Gene Ontology and Pathway Enrichment Analysis

To identify the biological functions associated with the differentially expressed genes (DEGs), gene ontology (GO) annotation and Kyoto Encyclopedia of Genes and Genomes (KEGG) pathway enrichment analysis were conducted using the database for annotation, visualization, and integrated discovery (DAVID) [19] version 6.8 with default parameters (*p* < 0.10). DEGs found in the β-alanine or L-histidine supplementation groups were independently subjected to GO enrichment and KEGG pathway analysis. The GO database comprises three main branches: biological process (BP), cellular component (CC), and molecular function (MF). 

### 2.6. Verification of RNA-Seq Results by Quantitative PCR (qPCR)

Differential expression results from RNA-Seq were confirmed by qPCR. The extracted RNA was reverse transcribed into cDNA using a High-Capacity cDNA reverse transcription kit (Thermo Fisher Scientific) following the manufacturer’s protocols. A total of five transcripts (i.e., *FOXO1*, *IGF2BP3*, *PDE10A*, *GRIP2*, and *PPM1K*) were randomly selected from DEGs that showed differences in both comparison groups, and primers were designed by applying NCBI Primer-BLAST on the website of NCBI (http://www.ncbi.nlm.nih.gov/tools/primer-blast/, accessed on 14 May 2021). β-Actin was used as an internal reference gene to normalize the expression data. The qPCR reactions were carried out on a LightCycler 480 System (Roche Diagnostics GmbH, Mannheim, Germany). The qPCR reactions had a total volume of 20 µL with 10 µL of SYBR Green I Master Mix (Roche Diagnostics GmbH), 1 µL of each primer (10 µM), 6 µL of nuclease-free water, and 2 µL of cDNA template. The thermocycling program consisted of initial denaturation at 95 °C for 5 min followed by 35 cycles of denaturation at 95 °C for 30 s, annealing at 61 °C for 30 s, and extension at 70 °C for 1 min for *FOXO1*, *PDE10A*, and *PPM1K*, or 40 cycles of denaturation at 95 °C for 30 s, annealing at 62 °C for 30 s, and extension at 72 °C for 1 min for *GRIP2* and *IGF2BP3*. All qPCR reactions were run with three replicates for each sample. Melting curve analysis was performed following the qPCR reactions. Relative gene expression values were calculated using the 2^−∆∆CT^ method [20]. The mean 2^−∆∆CT^ value for each gene was transformed into a fold change value to compare the qPCR results with those of RNA-Seq. The primer sequences used are listed in Table S2.

## 3. Results

### 3.1. Quality of RNA-Seq Reads 

An average of 46,769,860 raw sequencing reads were generated. After excluding low-quality reads, 15 high-quality samples were further mapped onto the chicken reference genome. The quality report for RNA-Seq showed that at least 94.31% of reads had a sequencing quality score exceeding Q30 (percentage of bases with a Phred value ≥ 30) in 15 libraries. The average ratio of high-quality reads to the reference genome was 82.43%. The results of quality assessment of sequencing data are presented in Table 1.

### 3.2. Significantly Differentially Expressed Transcripts

**Figure 1:**
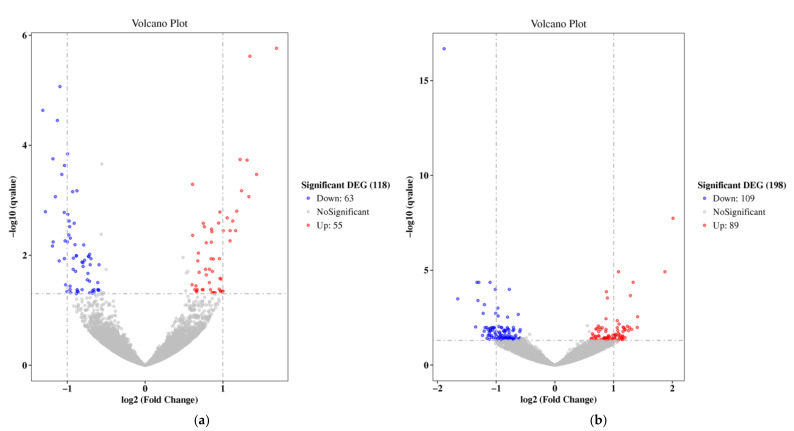
Scatter plot of differentially expressed genes: (**a**) volcano plot of the β-alanine supplementation; (**b**) volcano plot of the L-histidine supplementation.

Followin g RNA-Seq of the breast muscle of female KRCs in the non-supplemented group (A), β-alanine or L-histidine supplementation groups (B and C), we compared global gene expression in the B and C groups to that in group A so as to identify the underlying molecular mechanisms affected by diet. A total of 118 and 198 DEGs were identified in the groups B and C, respectively, compared to the group A (Figure 1). Compared with group A, group B had 55 upregulated genes and 63 downregulated genes, and group C had 89 upregulated genes and 109 downregulated genes among these DEGs. All information regarding the DEGs is provided in Supplementary Materials (Tables S3 and S4).

### 3.3. GO Annotation and KEGG Pathways Analyses of DEGs

The GO functional analysis identified 14 and 16 GO terms in the β-alanine or L-histidine supplementation groups, respectively (*p* < 0.10; Table S5), of which six and eight GO terms were significantly enriched (*p* < 0.05) (Table 2 and Table 3). No GO terms categorized into MF and CC branches were detected in the β-alanine or L-histidine supplementation groups. The identified KEGG pathways of the DEGs are listed in Table 4. Compared to group A, the insulin signaling pathway (gga04910) was identified as being enriched in the β-alanine supplementation group, whereas the insulin resistance (gga04910) and adipocytokine signaling (gga04920) pathways were enriched in the L-histidine supplementation group. The forkhead box O (FoxO) signaling pathway (gga04068) was commonly identified as a regulatory network in both dietary supplement groups. 

### 3.4. Validation of DEGs by qPCR

**Figure 2:**
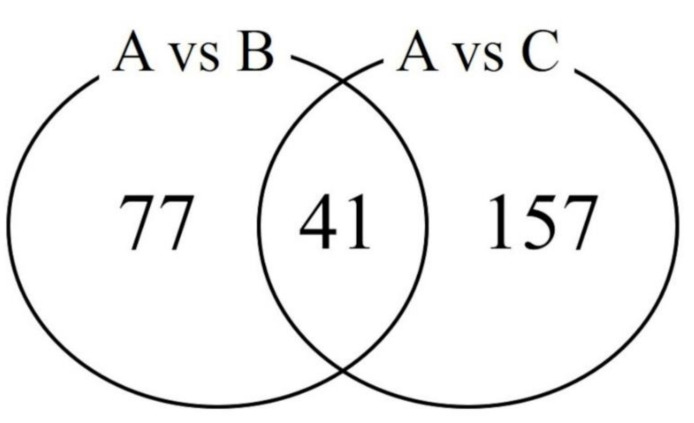
Number of differentially expressed genes in the β-alanine or L-histidine supplementation group compared to the non-supplemented group. A, B, and C represent the non-supplemented group, the β-alanine supplementation group, and the L-histidine supplementation group, respectively.

**Figure 3:**
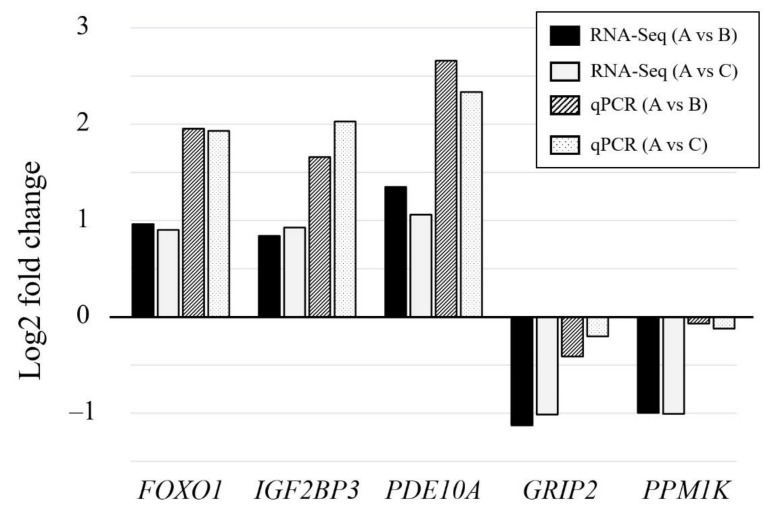
Validation of five DEGs detected from RNA-Seq analysis by qPCR. The *x*-axis represents the gene name and the *y*-axis represents the mRNA expression levels expressed in a fold change value. The expression levels obtained by RNA-Seq and qPCR are represented by solid fill and pattern fill columns, respectively. A, B, and C represent the non-supplemented group, the β-alanine supplementation group, and the L-histidine supplementation group, respectively. Gene abbreviation represents forkhead box O1 (*FOXO1*), insulin-like growth factor 2 mRNA binding protein 3 (*IGF2BP3*), phosphodiesterase 10A (*PDE10A*), glutamate receptor interacting protein 2 (*GRIP2*), and protein phosphatase Mg^2+^/Mn^2+^ dependent 1K (*PPM1K*), respectively.

Of the detected DEGs, 77 and 157 DEGs were only identified in the β-alanine or L-histidine supplementation groups, respectively, compared to the non-supplemented group (Figure 2). Forty-one genes were found to be common in the two comparison groups. Five DEGs, including three upregulated genes (*FOXO1*, *IGF2BP3*, and *PDE10A*) and two downregulated genes (*GRIP2* and *PPM1K*), were randomly selected from the 41 DEGs and their expression levels were verified by qPCR. The qPCR results showed that the expression trend of the DEGs was consistent in the RNA-Seq results in both comparison groups (Figure 3), suggesting that the RNA-Seq results in this study were accurate and reproducible.

## 4. Discussion

A previous study showed the breast meat from female KRCs fed diets without supplementation (A), supplemented with β-alanine (B) or L-histidine (C) contained 2756, 3484, and 3659 µg/g of carnosine, respectively, with shear force values of 3.31, 3.50, and 2.94 kg/g [7]. The carnosine content in group A and shear force value in group C were significantly lower than those in the other groups, respectively (*p* < 0.05) [7]. In this study, we performed RNA-Seq to identify differentially expressed genes and underlying molecular mechanisms affected by the diets supplemented with β-alanine or L-histidine. The quality report for RNA-Seq data obtained in this study (Table 1) indicated that the data were replicable and could be used in future analyses. In the differential expression analysis of transcripts (Figure 1, Tables S3 and S4), we identified some genes associated with myosin, collagen, intramuscular fat (IMF), and calpain, all of which may be related to meat quality.

Carnosine can be synthesized from β-alanine and L-histidine under in a reaction catalyzed by carnosine synthase. Increased skeletal carnosine concentrations in chickens by supplementation of dietary carnosine, β-alanine, or L-histidine have been reported elsewhere [5,6,21,22], and the upregulated expression of carnosine synthase corresponds with high carnosine content [6,21]. Qi et al. [6] reported that dietary supplementation with 250–2000 mg/kg β-alanine quadratically increased carnosine content, promoted mRNA expression of carnosine synthase 1 (*CARNS1*) and solute carrier family 6 member 6 (*SLC6A6*), and reduced the shear force of the breast muscle in broilers (*p* < 0.05). However, *CARNS1* and *SLC6A6* were not detected in this study. Carnosine content in chickens is strongly influenced by their breed [2]. Tian et al. [23] found that the carnosine content observed in the breast meat of Black-Bone Silky Fowl—a unique breed of chicken native to South China—was approximately 1.8-fold higher than that observed in the meat of White Plymouth Rock reared under the same conditions (*p* < 0.01). A recent study has reported significantly higher carnosine content in the breast muscles of Thai indigenous slow-growing chickens than in broiler chickens [24]. KRC is a crossbred chicken with Thai indigenous chickens and has a slow growth rate. The carnosine synthase gene was not detected as DEGs in this study, likely because slow-growing chickens have constantly high expression levels of this gene and are not affected by the supplemented diet. Downregulation of acidic amino acid decarboxylase GADL1 isoform X1 (*GADL1*), which is involved with β-alanine metabolism in chicken, was detected in the L-histidine supplementation group. GADL1 is considered to be an enzyme with multiple biological activities and is responsible for converting aspartic acid to β-alanine in carnosine biosynthesis [25]. In mice, *GADL1* knockout mice were deficient in β-alanine and carnosine in the skeletal muscle [25]. However, the association of the *GADL1* and carnosine contents has not been reported in chickens. It is still unclear how the downregulation of the *GADL1* gene by L-histidine supplementation contributes to an increase in the carnosine contents of female KRC breast muscle.

Muscle structure and composition (i.e., muscle fibers, intramuscular connective tissue, and intramuscular fat) in livestock are related to meat sensory quality, including tenderness [26]. The characterization of muscle fibers by contractile and metabolic properties suggested that the myosin heavy chain isoforms (MyHCs) existing in thick filaments has a significant effect on the muscle’s contractile properties. MyHCs appear to be the most appropriate markers for muscle fiber-type delineation [27]. Indeed, upregulation of myosin heavy chains 1 (*MYH1*) and 15 (*MYH15*) have been detected in tough meat in previous studies on beef cattle and chicken, respectively [11,28]. Although myosin-related genes were found to be downregulated in this study—i.e., VIIB (*MYO7B*) in the β-alanine supplementation group and myosin binding protein H like (*MYBPH*) in the L-histidine supplementation group—no differentially-expressed *MyHC* gene was identified in this study. In chickens, myosin binding protein H has been speculated to be involved in the regulation of muscle contraction [29]; however, its function remains unclear. The composition and structure of connective tissue also affect meat tenderness [30], and collagen is considered the primary determinant of the shear force, particularly in cattle. Piórkowska et al. [11] found that a gene encoding collagen protein or involved in their synthesis (i.e., *COL16A1*, *COL20A1*, *LEPREL4*, *PCOLCE2*, *P4HA3*, and *VWA1*) was highly expressed in chickens with lower levels of tenderness. This suggests that components of extracellular matrices, such as collagen, glycoproteins, and proteoglycans, are involved in determining the tenderness of broiler chicken meat. In our study, two genes related to collagen—serpin family H member 1 (*SERPINH1*), and collagen alpha-2(IV) chain-like (*LOC107051274*)—were downregulated and upregulated, respectively, in the β-alanine supplementation group. *SERPINH1* encodes Hsp47, a collagen-specific molecular chaperone. Hsp47 is essential for the molecular maturation of collagen, and Hsp47 expression strongly correlates with the expression of collagens in several types of cells and tissues [31]. Zhang et al. [32] reported upregulation of the *SERPINH1* gene in male Qinchuan cattle that had a significantly higher shear force than females. Downregulation of the *SERPINH1* was also detected in the present study; however, the shear force values did not differ significantly between groups supplemented and not supplemented with β-alanine. Although upregulation of the *LOC107051274* gene was detected after β-alanine supplementation, the function of this gene has not been reported yet. Future studies are required to clarify how altering the expression of these genes affects collagen synthesis in chickens. A certain amount of IMF can enhance meat quality traits in chickens [33]. For instance, Wen et al. [34] reported significant differences in tenderness between pectoral muscles with < 0.5% IMF and those with >0.5% IMF in partridges (*Alectoris chukar*) (*p* < 0.01). Several studies have reported DEGs related to IMF content in chickens [33,35]. Of these genes, upregulation of the acyl-CoA synthetase bubblegum family member 1 (*ACSBG1*) gene was detected in the L-histidine supplementation group. Although this gene mediates the activation of long-chain fatty acids to synthesize cellular lipids [35], a study on IMF deposition is required to clarify the involvement of this gene in KRCs.

Shear force depends not only on the structural and metabolic properties of a muscle at the time of slaughter, but also on muscle metabolism during rigor mortis and aging [34,36]. The kinetics of postmortem muscle acidification are faster in poultry than in cattle and strongly affect tenderness of the meat [26]. Calpain is an intracellular Ca^2+^-dependent cysteine protease that is closely related to the tenderness of meat in livestock [37]. Okumura et al. [38] identified four calpain genes (i.e., *CAPN1*, *CAPN2*, *CAPN3*, and *CAPN1.5*) that were ubiquitously expressed in chickens. Among these, a single nucleotide polymorphism (SNP) in the *CAPN1* gene was significantly related to meat tenderness in chickens and Japanese quail [10,39]. Moreover, Piórkowska et al. [40] found that higher expression of the *CAPN1* gene leads to higher tenderness in breast muscles in fast-growing broiler chickens. The *CAPNS1* and *CAPNS2* genes encode the small subunits one and two of calpain, respectively [41], and upregulation of the *CAPNS2* gene was detected in the β-alanine supplementation group in this study. Although the *CAPNS1* protein is known to be important for maintaining the stability of both conventional catalytic subunits of calpain, its physiological role is yet unknown [41]. Recently, a significant association has been reported between SNPs in *CAPNS2* and *CAPN1* genes and meat tenderness in Iberian pigs, and the G allele in *CAPN1_rs8135866G > A* SNP was associated with tender meat [42]. These results suggest that the calpain enzyme is related to meat tenderness in chickens and the upregulation of the *CAPNS2* gene by β-alanine supplementation may associate with meat toughness.

The GO analysis showed that compared to the non-supplemented group, the DEGs were significantly enriched in six and eight functions in the β-alanine or L-histidine supplementation groups, respectively (*p* < 0.05, Table 2 and Table 3). These functions did not overlap between the groups. Among the DEGs that may be related to meat tenderness (i.e., *MYO7B*, *MYBPH*, *LOC107051274*, *SERPINH1*, *ACSBG1*, and *CAPNS2*), *SERPINH1* was identified in the “extracellular space” term of the CC category, along with another 11 DEGs (Table 2). Although this gene is associated with collagen, no BP terms involving collagen were detected in either experimental group. Listrat et al. [26] reported that collagen has a limited effect on the sensory quality of chicken meat because the animals are slaughtered at an early physiological stage when intramuscular collagen is not cross-linked significantly. El-Senousey et al. [43] reported that dietary alpha-lipoic acid significantly decreased collagen content and mRNA expression of the *COL3A1* gene in broiler chickens, although it did not change meat tenderness. Katemala et al. [44] reported that the breast meat of 10-week-old KRC exhibited higher shear force values than that of commercial broiler chickens (*p* < 0.05), although there was no difference in the collagen content. These findings suggest that the higher tenderness detected in the L-histidine supplementation group was not caused by changes in the extracellular matrix (e.g., due to changes in collagen synthesis). Of the BP terms listed in Table 3, wound healing and gluconeogenesis were also identified among the top 10 enriched GO terms in a transcriptome study on the breast muscles of two broiler chicken lines that showed significant differences in meat quality (ultimate pH, muscle glycolytic potential, and toughness) [45]. In this study, we found that the L-histidine supplementation group showed downregulation of the 6-phosphofructo-2-kinase/fructose-2,6-biphosphatase 1 and phosphoglycerate mutase 1 (*PGAM1*) genes, upregulation of the cryptochrome circadian regulator 1 gene, and enrichment in the gluconeogenesis function. A high correlation has been reported between three proteins—pyruvate kinase, PGAM1, and triosephosphate isomerase 1—and the tenderness of breast meat in Thai indigenous chickens [46]. Moreover, significantly higher levels of these three proteins have also been observed in the tough meat. PGAM catalyzes the interconversion of 2-phosphoglycerate and 3-phosphoglycerate in the glycolytic cycle [47]. These findings suggest that the DEGs involved in gluconeogenesis are associated with meat characteristics and that the *PGAM1* gene can be a functional marker for meat tenderness in KRC.

The KEGG pathway analysis comparing the non-supplemented and supplemented groups identified the insulin signaling pathway in the β-alanine-supplemented group and insulin resistance and adipocytokine signaling pathway in the L-histidine-supplemented group (*p* < 0.05, Table 4). In animals, adipogenesis occurs earliest in visceral fat deposits, followed by adipogenesis in subcutaneous, intermuscular, and intramuscular fat deposits [48]. Insulin affects a series of biological reactions in muscle and adipose tissues, including lipid metabolism [49]. By comparing DEGs between muscle and fat tissues in broiler chickens, Zhang et al. [50] identified lipid metabolism signaling pathways involved in the regulation of fat deposition (i.e., adipocytokine signaling pathway, insulin signaling pathway, and pyruvate metabolism). These findings suggest that the insulin-related and adipocytokine signaling pathways affected by the supplemented diet likely regulate fat deposition in intermuscular adipose tissue in the breast muscle of KRC. In this study, the FoxO signaling pathway was identified as a common regulatory network affected by the supplemented diet. FoxO acts as a regulator to maintain tissue homeostasis over time and affects many cellular processes involved in apoptosis, cell cycle regulation, DNA damage repair, metabolism, and oxidative stress [51,52]. The FoxO family consists of four members—i.e., FOXO1, FOXO3, FOXO4, and FOXO6—and upregulation of the *FOXO1* gene was observed in this study. FoxOs have been previously identified as potential candidate genes regulating growth in chickens [53,54]. Willson et al. [55] investigated gene expression in the liver among meat birds, the layer strain, and their F1 crosses, and found that the FoxO signaling pathway—through its effects on cell regulation and altered metabolism—is associated with growth variations in chickens. Furthermore, Li et al. [56] studied miRNA and mRNA expression in slow-growing chickens at 6, 14, 22, and 30 weeks of age and found that FoxO signaling is a key pathway underlying the postnatal development of breast muscles in slow-growing chickens. Postnatal growth of skeletal muscle is achieved primarily by increasing the hypertrophy of existing muscle fibers [56]. This suggests that the FoxO signaling pathway is related to muscle fiber hypertrophy. FoxO transcription factors act as negative regulators of type I fiber-related gene expression in muscle atrophy [57]. The thigh muscles of broiler chickens fed a high-carnosine diet show a higher density of type I fiber and higher carnosine content along with a decrease in type I fiber diameter and meat toughness (*p* < 0.05) [21]. These findings suggest that the expression levels of FoxO transcription factors may correlate with muscle fiber size after diet supplementation with β-alanine or L-histidine. In broiler, increased skeletal muscle fiber diameter and area could increase shear force and reduce meat tenderness [58]. A significant association has been reported between the genotype of the *CAPN1* gene and breast muscle fiber diameter in KRC, and KRCs carrying the BB genotype showed a significantly smaller muscle fiber diameter at 10 weeks of age [59]. Genetic selection of female KRCs for large muscle fiber size combined with feeding them diets supplemented with β-alanine or L-histidine may produce meat containing high carnosine concentrations without losing the toughness unique to this meat.

## 5. Conclusions

This study analyzed differences in global gene expression in the breast muscle of female KRCs with differing carnosine contents and meat toughness that were produced by dietary supplementation with β-alanine or L-histidine. Compared to the non-supplemented group, 118 and 198 DEGs were identified in the β-alanine or L-histidine supplementation groups, respectively. Although dietary supplementation with β-alanine or L-histidine produced breast meat with significantly higher carnosine content, genes previously reported to be associated with increasing carnosine content, such as *CARNS1*, were not activated. Functional enrichment analysis of the DEGs identified different underlying genetic mechanisms that were altered by each supplemented diet and likely regulated meat tenderness. The identified genes, including *MYO7B*, *MYBPH*, *LOC107051274*, *SERPINH1*, *ACSBG1*, *CAPNS2*, and *PGAM1,* may be potential functional markers for meat tenderness in slow-growing chickens. However, we did not examine the structural and metabolic properties of a muscle in this study, future studies are required to clarify how altering the expression of these genes affects meat tenderness.

## Figures and Tables

**Table 1:** RNA-Seq reads and mapping rate of breast muscle samples from 15 chickens fed an experimental diet.

Sample ID ^1^	Raw Reads	Clean Reads	Q30 (%) ^2^	GC Content (%)	Total Mapped Read	Uniquely Mapped Reads	Multiple Mapped Reads	Mapping Rage (%)
A1	46,462,620	46,288,554	94.94	55.01	37,652,127	33,451,212	4,200,915	81.34
A2	47,166,518	47,013,356	95.04	54.01	39,871,968	35,898,964	3,973,004	84.81
A3	47,881,994	47,713,850	94.31	56.61	36,339,766	32,824,893	3,514,873	76.16
A4	43,994,624	43,820,892	94.49	56.25	33,585,942	28,618,863	4,967,079	76.64
A5	48,893,704	48,746,180	95.02	54.87	41,053,319	35,969,263	5,084,056	84.21
B1	46,163,116	46,008,446	94.76	54.55	38,295,423	34,456,408	3,839,015	83.24
B2	48,282,906	48,121,860	94.81	55.03	40,616,995	35,710,664	4,906,331	84.4
B3	47,914,044	47,776,800	94.63	55.36	39,450,436	36,438,554	3,011,882	82.57
B4	47,830,614	47,678,704	94.66	54.1	39,195,229	35,730,602	3,464,627	82.21
B5	46,867,322	46,705,710	94.61	54.5	38,611,389	35,737,411	2,873,978	82.67
C1	46,346,778	46,172,558	94.8	53.96	38,610,455	35,584,466	3,025,989	83.62
C2	46,252,972	46,087,264	94.89	53.78	39,318,314	36,417,362	2,900,952	85.31
C3	47,149,780	47,007,510	94.36	54.77	38,372,185	34,911,827	3,460,358	81.63
C4	48,618,940	48,452,690	95.0	54.46	40,454,304	36,297,707	4,156,597	83.49
C5	41,721,980	41,582,684	94.67	53.52	35,046,741	32,462,385	2,584,356	84.28
Average	46,769,860.8	46,611,803.8	94.73	54.71	38,431,639.5	34,700,705.4	3,730,934.1	82.43

^1^ Sample ID represents treatment group and biological replicate number. Treatment groups are A (non-supplemented), B (β-alanine supplementation), and C (L-histidine supplementation), respectively. A1, A2, A3, A4, and A5 indicate the five biological replicate breast muscle tissues in treatment group A, the rest of the groups (B and C) share the same name rules. ^2^ Q30 indicates the percentage of bases with a Phred value ≥ 30.

**Table 2:** Significantly enriched gene ontology terms in the β-alanine supplementation group compared to the non-supplemented group.

Category	Term ID	Term	Count	*p*-Value	Genes ^1^
Biological process	GO:0003151	Outflow tract morphogenesis	4	6.75 × 10^−4^	*BMP4↑, HEYL↓, DHRS3↑, SFRP2↓*
	GO:0007160	Cell-matrix adhesion	3	0.031	*OTOA↑, TSC1↑, ITGB1BP1↑*
	GO:0002043	Blood vessel endothelial cell proliferation involved in sprouting angiogenesis	2	0.034	*BMP4↑, ITGB1BP1↑*
Cellular component	GO:0009986	Cell surface	8	7.13 × 10^−4^	*ADAMTS15↓, RTN4RL1↓, SDC1↓, CFTR↑, VASN↓, PLA2R1↑, OTOA↑, THBD↓*
	GO:0005615	Extracellular space	12	0.001	*PXDNL↓, ADAMTS15↓, TST↓, BMP4↑, PLA2G15↑, VASN↓, TNFSF10↑, CPN1↓, SERPINH1↓, SFRP2↓, PPFIBP2↑, CTSV↑*
	GO:0016324	Apical plasma membrane	4	0.029	*CNTFR↓, CFTR↑, OTOA↑, AMOTL1↑*

^1^ Genes with up and down arrows represent those upregulated or downregulated in the supplementation group, respectively.

**Table 3:** Significantly enriched gene ontology terms in the L-histidine supplementation group compared to the non-supplemented group.

Category	Term ID	Term	Count	*p*-Value	Genes ^1^
Biological process	GO:0035556	Intracellular signal Transduction	10	5.68 × 10^−4^	*ASB8↓, ASB2↑, SGK1↑, PRKAA2↓, RGS9↑, ASB5↑, NUAK1↑, DAPK1↑, SPSB1↑, ARHGEF7↑*
	GO:0042752	Regulation of circadian rhythm	3	0.025	*PRKAA2↓, NOCT↓, PPARA↑*
	GO:0006094	Gluconeogenesis	3	0.032	*PFKFB4↓, PGAM1↓, CRY1↑*
	GO:0010629	Negative regulation of gene expression	4	0.038	*CDKN1A↓, CTGF↑, TIPARP↓, NOCT↓*
	GO:0009267	Cellular response to Starvation	3	0.039	*PRKAA2↓, MYOD1↑, PIK3C2B↓*
	GO:0042060	Wound healing	3	0.039	*PECAM1↑, PPARA↑, SLC11A1↓*
	GO:0032922	Circadian regulation of gene expression	3	0.045	*NOCT↓, PPARA↑, CRY1↑*
Molecular function	GO:0004722	Protein serine/threonine phosphatase activity	3	0.029	*PPM1K↓, PPM1J↓, PDP1↓*

^1^ Genes with up and down arrows represent those upregulated or downregulated in the supplementation group, respectively.

**Table 4:** Significantly enriched KEGG pathway in the β-alanine or L-histidine supplementation groups compared to the non-supplemented group.

Term	Count	*p*-Value	Genes ^1^
β-alanine supplementation			
gga04068: FoxO signaling pathway	5	0.008	*FOXO1*↑, *CCNG2*↑, *TNFSF10*↑, *G6PC3*↓, *IRS2*↑
gga04910: Insulin signaling pathway	4	0.044	*FOXO1*↑, *G6PC3*↓, *IRS2*↑, *TSC1*↑
L-histidine supplementation			
gga04068: FoxO signaling pathway	6	0.008	*CDKN1A*↓, *SGK1*↑, *PRKAA2*↓, *FOXO1*↑, *TNFSF10*↑, *IRS2*↑
gga04931: Insulin resistance	5	0.018	*PRKAA2*↓, *FOXO1*↑, *PPARA*↑, *CREB5*↑, *IRS2*↑
gga04920: Adipocytokine signaling pathway	4	0.029	*ACSBG1*↑, *PRKAA2*↓, *PPARA*↑, *IRS2*↑

^1^ Genes with up and down arrows represent that upregulated or downregulated in the supplementation group, respectively.

## Data Availability

Raw sequencing data are accessible through GEO Series accession number GSE179643 (https://www.ncbi.nlm.nih.gov/geo/query/acc.cgi?acc=GSE179643). All data generated or analyzed during this study available from the corresponding authors on reasonable request.

## Supplementary Materials

The following are available online at https://www.mdpi.com/article/10.3390/ani11092596/s1, Table S1: Composition of the experimental diet, Table S2: List of primer sequences for quantitative PCR, Table S3: Differentially expressed genes in the β-alanine supplementation group compared to the non-supplemented group, Table S4: Differentially expressed genes in the L-histidine supplementation group compared to the non-supplemented group, Table S5: Gene ontology terms for the differentially expressed genes in the β-alanine or L-histidine supplementation groups compared to the non-supplemented group.
