# Supplementary material for: RNA Profiles of the Korat Chicken Breast Muscle with Increased Carnosine Content Produced through Dietary Supplementation with β-Alanine or L-Histidine
Source: Animals (Basel). 2021 Sep 3;11(9):2596. doi: 10.3390/ani11092596 (PMC8464878; doi:10.3390/ani11092596)
Supplement: Supplementary file 1 [file animals-11-02596-s001.zip › TableS1.pdf]

Table S1. Composition of the experimental diet.

	Grower (22 to 42 days)			Finisher (43 to 70 days)		
Treatment group[1]	A	B	C	A	B	C
Ingredients (g/100 g)						
Corn	59.4	57.52	58.3	67.3	65.14	66.14
Soybean meal (44% CP)	30.8	28.4	28.22	26.3	26.7	26.5
Full fat soybean	2.42	5.5	5.8	0	0	0
Rice bran oil	4	4.2	3.8	3.04	3.8	3.5
L-Lysine	0.18	0.18	0.18	0.19	0.19	0.19
DL-Methionine	0.21	0.21	0.21	0.14	0.14	0.14
Salt	0.35	0.35	0.35	0.35	0.35	0.35
Calcium carbonate	1.42	1.42	1.42	1.2	1.2	1.2
MDCP (P21)	1.02	1.02	1.02	1.28	1.28	1.28
Premix[2]	0.2	0.2	0.2	0.2	0.2	0.2
β-Alanine	0	1	0	0	1	0
L-Histidine	0	0	0.5	0	0	0.5
Calculated composition (%)						
ME (kcal/kg)	3,113	3,116	3,112	3,112	3,116	3,118
Crude protein, %	19.39	19.30	19.39	17.08	17.10	17.08
Crude fiber, %	3.6	3.56	3.58	3.32	3.3	3.31
Ether extract, %	6.94	7.6	7.29	5.81	6.49	6.23
Calcium, %	0.9	0.9	0.9	0.86	0.86	0.86
Total phosphorus, %	0.57	0.57	0.57	0.6	0.59	0.59
Histidine	0.45	0.48	0.99	0.37	0.37	0.87

[1] Treatment groups are A (without supplementation), B (supplemented with 1% alanine), and C (supplemented with 0.5% L-histidine), respectively.

[2] Premix (0.5%) provided the following per kilogram of diet: 15,000 IU of vitamin A; 3000 IU of vitamin D3; 25 IU of vitamin E; 5 mg of vitamin K3; 2 mg of vitamin B1; 7 mg of vitamin B2; 4 mg of vitamin B6; 25 ug of vitamin B12; 11.04 mg of pantothenic acid; 35 mg of nicotinic acid; 1 mg of folic acid; 15 ug of biotin; 250 mg of choline chloride; 1.6 mg of Cu; 60 mg of Mn; 45 mg of Zn; 80 mg of Fe; 0.4 mg of I; 0.15 mg of Se.
