# Supplementary material for: RNA Profiles of the Korat Chicken Breast Muscle with Increased Carnosine Content Produced through Dietary Supplementation with β-Alanine or L-Histidine
Source: Animals (Basel). 2021 Sep 3;11(9):2596. doi: 10.3390/ani11092596 (PMC8464878; doi:10.3390/ani11092596)
Supplement: Supplementary file 1 [file animals-11-02596-s001.zip › TableS2.pdf]

Table S2. List of primer sequences for quantitative PCR.

Gene[1]	Primer sequence (5'-3')	Product size (bp)	GenBank accession number
FOXO1	F: ACAGTTGGGTGTCAGGCTAGG	139	NM_204328.1
	R: ATGCGCACAGCTTTGTCAAGA		
IGF2BP3	F: TCCTGCAGTGAGTGTTGCCT	103	NM_001006359
	R: TGCCAAGGCTGGGATGAACA		
PDE10A	F: AAGATCTCGCAAGCCACACG	72	XM_004935554.3
	R: TGCATCCATGAGCCCCTTCTC		
GRIP2	F: ACACGGTGGAACTGAAGCGA	139	XM_040646724.1
	R: TCACCGATGTGGATGGCTCC		
PPM1K	F: GCCCTGCTGTGTCGAAAAGG	164	XM_420574.6
	R: CCTATGCTCCGCGTCATTGC		
ACBT	F: TGACCGCGTTACTCCCACAG	90	NM_205518.1
	R: CGAAACCGGCCTTGCACATA		

[1] Gene abbreviation represents forkhead box O1 (*FOXO1*), insulin-like growth factor 2 mRNA binding protein 3 (*IGF2BP3*), phosphodiesterase 10A (*PDE10A*), glutamate receptor interacting protein 2 (*GRIP2*), and protein phosphatase Mg2+/Mn2+ dependent 1K (*PPM1K*), respectively.
